# A new sign in paracoccidioidomycosis neuroimaging

**DOI:** 10.1590/0100-3984.2023.56.4e4-en

**Published:** 2023

**Authors:** José Daniel Vieira de Castro

**Affiliations:** 1 Full Professor of Radiology, School of Medicine, Universidade Federal do Ceará (UFC), Fortaleza, CE, Brazil.Email: danielcastrorevisor@gmail.com

Paracoccidioidomycosis (PCM) is a systemic fungal disease caused by *Paracoccidioides brasiliensis* or *Paracoccidioides lutzii*^(1)^. Two main clinical forms of PCM have been described^(2)^: the juvenile (acute/subacute) form; and the adult (chronic) form. The chronic form is an endemic disease in South and Central America, predominantly affecting rural workers, typically between 30 and 50 years of age, with a clear predominance of males. In Brazil, PCM occurs mainly in the states of São Paulo, Rio de Janeiro, Minas Gerais, Rio Grande do Sul, and Mato Grosso^(3)^. It is acquired by inhaling the conidia produced in the mycelial form, which transforms into the yeast form, classically causing a benign pulmonary infection. The disease can then spread to other organs through the blood or lymphatic system^(4)^. It is more common in immunocompetent hosts. A small percentage of cases occur in immunocompromised patients, the vast majority of those cases having been reported in HIV-infected patients^(5)^.

In recent decades, with the development of neuroimaging, it has been determined that the involvement of the central nervous system (CNS) in PCM is much more common than previously thought, occurring in up to 36% of cases^(6)^. Two main forms of neuroparacoccidioidomycosis (NPCM) have been described^(7,8)^: a meningeal form; and a pseudotumoral form. The pseudotumoral form is the most common, presenting as single or multiple granulomas in the brain parenchyma or spinal cord^(9)^. The radiological findings are quite variable. On computed tomography, the pattern most commonly described is that of a hypodense lesion with annular enhancement, which is not very helpful in the etiological diagnosis because it resembles many granulomatous CNS diseases and even some brain neoplasms. The presence of calcified lesions with annular enhancement or multilocular lesions is more helpful in the diagnosis of NPCM, although those patterns are less common. On magnetic resonance imaging (MRI), the main findings are a hyperintense signal on T1-weighted images and a hypointense signal on T2-weighted images. Peripheral enhancement, in a “ring enhancement” pattern, is the most consistent pattern on contrast-enhanced T1-weighted images. Nodular, heterogeneous, leptomeningeal and pachymeningeal patterns of contrast enhancement are also seen. More recently, aspects of NPCM on imaging that employs advanced quantitative MRI techniques, such as diffusion-weighted imaging, susceptibility-weighted imaging, proton MR spectroscopy, and perfusion MRI, have been described. Restricted diffusion has been identified in less than 50% of cases. On proton MR spectroscopy, lipid peaks have been detected. On susceptibility-weighted imaging, the dual rim sign, similar to that seen in cases of pyogenic brain abscess, has been described. Therefore, even the new techniques have produced findings that are nonspecific and overlap with those of other CNS diseases^(6,10)^. Consequently, in endemic areas such as Brazil, NPCM should be considered in the differential diagnosis of a ring-enhanced mass seen on neuroimaging^(8,9)^. Due to the lack of specificity of imaging methods, brain biopsy is still the most widely used method for the definitive diagnosis of NPCM^(6)^.

It remains unclear what should be the treatment of choice for NPCM, given that there is considerable heterogeneity among studies in terms of the treatment employed. The drugs most commonly used are trimethoprim-sulfamethoxazole and amphotericin B. Even with treatment, approximately 76% of patients have sequelae, and a delay in the start of treatment increases the incidence of such sequelae^(6)^.

The article *“Star of Bethlehem sign” in the analysis of the evolution of brain lesions during and after treatment for neuroparacoccidioidomycosis*, authored by Santana et al.^(11)^ and published in this issue of Radiologia Brasileira, despite being a retrospective analysis, makes a significant contribution by taking a different approach from other works, providing, for the first time, an analysis of the evolution of the imaging aspects of NPCM on post-treatment MRI of 56 brain lesions. In their study, the authors found that 84.4% of the lesions smaller than 1.2 cm disappeared during treatment, whereas the larger lesions tended to remain stable, in terms of size and peripheral enhancement, even after treatment. This suggests that early institution of treatment improves the prognosis. In addition, the authors describe an interesting finding, which they named the “Star of Bethlehem sign”, characterized by an eccentric mural nodule, with contrast enhancement, observed in lesions larger than 1.2 cm and disappearing during treatment. Its disappearance could be a marker of lesion inactivity, given that the other aspects (size and peripheral enhancement) persisted over time, even after the end of treatment. Further prospective studies involving post-treatment biopsy of these lesions might come to corroborate that hypothesis.
